# Chest pain in the ambulance; prevalence, causes and outcome - a retrospective cohort study

**DOI:** 10.1186/s13049-019-0659-6

**Published:** 2019-08-29

**Authors:** Claus Kjær Pedersen, Carsten Stengaard, Kristian Friesgaard, Karen Kaae Dodt, Hanne Maare Søndergaard, Christian Juhl Terkelsen, Morten Thingemann Bøtker

**Affiliations:** 1 Department of Cardiology, Aarhus University Hospital, Palle Juul-Jensens Boulevard 99, 8200 Aarhus N, Denmark; 2 Department of Anesthesiology, Aarhus University Hospital, Aarhus, Denmark; 3 Research and Development, Prehospital Emergency Medical Services, Central Denmark Region, Aarhus, Denmark; 4 Department of Internal Medicine, Regional Hospital Horsens, Horsens, Denmark; 5 Department of Cardiology, Regional Hospital Viborg, Viborg, Denmark

**Keywords:** ACS, AMI, Chest pain, Prehospital diagnosis, Prehospital triage, EMS

## Abstract

**Background:**

Chest pain is common in acute ambulance transports. This study aims to characterize and compare ambulance-transported chest pain patients to non-chest pain patients and evaluate if patient characteristics and accompanying symptoms accessible at the time of emergency call can predict cause and outcome in chest pain patients.

**Methods:**

Retrospective, observational population-based study, including acute ambulance transports. Patient characteristics and symptoms are included in a multivariable risk model to identify characteristics, associated with being discharged without an acute cardiac diagnosis and surviving 30 days after chest pain event.

**Results:**

In total, 10,033 of 61,088 (16.4%) acute ambulance transports were due to chest pain. In chest pain patients, 30-day mortality was 2.1% (95%CI 1.8–2.4) compared to 6.0% (95%CI 5.7–6.2) in non-chest pain patients. Of chest pain patients, 1054 (10.5%) were diagnosed with acute myocardial infarction, and 5068 (50.5%) were discharged without any diagnosis of disease. This no-diagnosis group had very low 30-day mortality, 0.4% (95%CI 0.2–0.9). Female gender, younger age, chronic pulmonary disease, absence of accompanying symptoms of dyspnoea, radiation, severe pain for > 5 min, clammy skin, uncomfortable, and nausea were associated with being discharged without an acute cardiac diagnosis and surviving 30 days after a chest pain event.

**Conclusion:**

Chest pain is a common reason for ambulance transport, but the majority of patients are discharged without a diagnosis and with a high survival rate. Early risk prediction seems to hold a potential for resource downgrading and thus cost-saving in selected chest pain patients.

**Electronic supplementary material:**

The online version of this article (10.1186/s13049-019-0659-6) contains supplementary material, which is available to authorized users.

## Introduction

Chest pain is one of the most frequent symptoms among patients contacting the emergency medical services (EMS) and emergency departments (EDs) [1, 2]. In the United States, the estimated number of ED contacts due to chest pain exceeded 7 million in 2015 [3, 4]. Health care expenses for handling these patients are massive and are mainly driven by length of hospital stay [5]. Depending on the underlying cause, an acute EMS-response and subsequent hospitalization may prove relevant or non-relevant in chest pain patients.

Since chest pain is associated with potential life threatening conditions like acute myocardial infarction (AMI), pulmonary embolism or acute aortic dissection, the EMS response is most often dispatched in an “all or nothing”-fashion with a high triage level and immediate response. This level of triage is relevant in patients with a subsequent confirmed serious/adverse diagnosis and may favour an improved outcome [6, 7]. While patients with AMI often present with chest pain, the majority of patients presenting with chest pain may suffer from other more harmless conditions [8–10]. A mismatch like this could lead to overtriage and an excess of non-relevant EMS responses in chest pain patients. Secondarily this leads to high EMS-resource consumption, crowding, and prolonged length-of-stay (LOS) in EDs and coronary care units (CCUs) [2, 8, 11].

Consequently, there is a need for evaluating prehospital over-triage and non-relevant EMS use in chest pain patients by examining diagnoses and mortality rates in patients presenting with chest pain and a need for addressing ways of early discrimination between high- and low-risk patients and thus support the decision of when an acute EMS response is relevant or may be down-graded. Structured use of patient characteristics and presence/absence of accompanying symptoms could be a key factor in improving the triage in chest pain patients [12]. Therefore, we aimed to compare EMS-patients according to presence of chest pain and to identify predictors of acute cardiac conditions and death.

## Methods

### Aim

The specific aims of the present study were, in patients transported to hospital by ambulance:

- To describe the prevalence of chest pain and compare patient characteristics, mortality rates, diagnostic patterns, LOS, and place of primary admittance between chest pain and non-chest pain patients
- To compare mortality rates and LOS among chest pain patients according to the final diagnoses
- To identify predictors (available at the time of ambulance dispatch) of “potential for downgrading EMS response” in chest pain patients, indicated by discharge without an acute cardiac diagnosis and survival for more than 30 days after chest pain event.

### Study design and setting

This retrospective, observational population-based study was approved by The Danish Data Protection Agency, Central Denmark Region (ref. number 1–16–02-788-17) and the Danish Patient Safety Authority (ref. number 3–3013-2321).

The Danish health care system, including the EMS system, has previously been described [13, 14]. In Denmark EMS assistance can be requested either via medical emergency call (1-1-2) or by request from a general practitioner (GP) both during day-time and out-of-hours. When EMS is requested via 1-1-2, the medical emergency/injury is triaged according to the symptom/criteria-based Danish Index for Emergency Care [15]. However, when ambulance dispatch is requested by a GP, the prehospital response is based on the GPs’ clinical assessment rather than dispatch criteria [14].

### Data collection and processing

We collected dispatch information and information on the preceding 1-1-2 call and merged these with data from the Danish National Patient Registry to include data on hospital admissions and existing comorbidity [16]. We acquired vital status from the public Danish Civil Registration System. The use of the Danish health registries in research has previously been validated [17, 18].

### Selection of participants

This study included patients transported to hospital by ambulance in the Central Denmark region in a 1-year period from May 1st 2015 to April 30th 2016. We included patients transported after 1-1-2 call and patients transported following request from GP. We excluded transports missing information on symptoms or index diagnosis and transports terminated before reaching hospital, see Additional files 1 and 2.

### Variables

Way of entrance into the EMS system was categorized as “112-requested” or “GP-requested”. Chest pain was identified through dispatch codes for patients undergoing criteria-based dispatch (112-requested) and through specific text searches in electronic prehospital patient files (in GP-requested transports). The automatic text search to identify chest pain/discomfort in the patient files was verified by manual review of 3500 patient files (5%). Details on the automatic text search and validation are described in Additional file 3. We retrieved ICD-10 diagnoses given during index admission to identify final diagnosis of index admission, and within 10 years prior to the admission date to calculate Charlson Comorbidity Index (CCI), and to identify known comorbidity, see Additional file 4 [19].

We categorized final diagnosis according to the ICD-10 codes assigned to the patient as primary diagnoses during the admission following the EMS transport (=index admission):

1. Acute cardiac conditions (AMI and other acute/potentially life-threatening cardiovascular diagnoses)
2. Other diagnoses according to ICD-10 chapters
3. No final diagnosis (ICD-10 diagnosis of “Rxx.x”, “Z03.x” or “Z04.x” AND none of the above mentioned during index admission)

See Additional file 4 for specific ICD-10 codes and categories and exceptions in category 3.

### Outcomes

Primary outcome: Death within 30 days of admission.

Secondary outcome: Acute cardiac diagnosis during index admission

Composite secondary endpoint: To investigate if the ambulance transport and the succeeding hospital admissions in chest pain patients were appropriate or not, we constructed a combined secondary outcome of being discharged without an acute cardiac condition and surviving more than 30 days after chest pain event.

See Additional file 4 for specific ICD 10 codes.

### Statistical methods

Categorical data are presented as numbers and proportions. Continuous data are presented as means with 95% confidence interval (CI) or medians and interquartile ranges (IQR). For comparison between groups, Fisher’s exact test and Pearson’s chi-squared test were used for categorical data, and unpaired samples T-test and Wilcoxon Rank Sum for continuous data as appropriate. Mortality is described as Kaplan-Meier curves truncated at 30 days. Follow-up was terminated November 14th 2016 and only first transport registered for each patient was included into mortality analysis, see flowchart in Additional file 1.

We performed two predefined multivariable prediction models, using binary regression analyses in patients with chest pain, to evaluate the association between the secondary composite endpoint of not being diagnosed with an acute cardiac condition and surviving more than 30 days and different patient characteristics and accompanying symptoms – information that is potentially available at the time of emergency call. The first model, applied to all patients suffering chest pain, included: 1) age, gender, prior AMI, diabetes mellitus, known congestive heart disease, chronic pulmonary disease, and moderate to severe renal disease. The second model included the same patient characteristics as in model 1, but also accompanying symptoms. This was systematically registered only for those patients undergoing criteria-based dispatch (not GP requested dispatch) and thus, this model was applied to that sub-group only.

Age was included as age-groups (<=29, 30–49, 50–59, 60–69, ≥70 years). The mentioned predictor variables were included in multivariable analysis.

Regression models were evaluated 1) by plotting predicted and observed frequencies against age group as relevant in subgroups according to the remaining variables, 2) by likelihood-ratio test, and 3) by calculation of goodness-of-fit. All calculations were two-sided and *P*-values < 0.05 were considered statistically significant. Analyses were performed using STATA Intercooled software, version 15.1.

## Results

### Characteristics of study subjects

We identified 71,891 acute ambulance transports in the prehospital dispatch system during the study period, see flowchart in Additional file 1. After exclusion of patients not transported to hospital and patients with no available description of symptoms and final diagnosis, we included 61,088 transports. We analysed mortality in 47,601 unique first patient contacts. Patient and transport characteristics are summarized in Table 1.

**Table 1:** Baseline characteristics, prevalence of chest pain, incidence of AMI and other diagnoses among all GP- and 1–1-2 triaged patients

	All transports	Chest pain		*p*-value
		No	Yes	
Acute ambulance transports, n(%)	61,088 (100.0%)	51,055 (83.6%)	10,033 (16.4%)	
Registrations, N per 1000 inhabitants per year	47.5	39.7	7.8	
Way of ambulance request, n(%)				
GP	32,036 (52.4%)	25,743 (50.4%)	6293 (62.7%)	< 0.001
112	29,052 (47.6%)	25,312 (49.6%)	3740 (37.3%)	
Pre-hospital triage, n(%)				
A	28,230 (46.3%)	19,420 (38.1%)	8810 (87.8%)	< 0.001
B	24,754 (40.6%)	23,813 (46.7%)	941 (9.4%)	
C	8046 (13.2%)	7766 (15.2%)	280 (2.8%)	
Male gender, n(%)	31,563 (51.7%)	26,005 (50.9%)	5558 (55.4%)	< 0.001
Age, median (IQR)	65 (45, 78)	65 (42, 78)	66 (52, 77)	< 0.001
Diabetes, n(%)	7080 (11.6%)	5754 (11.3%)	1326 (13.2%)	< 0.001
Prior AMI, n(%)	4273 (7.0%)	2718 (5.3%)	1555 (15.5%)	< 0.001
Charlson comorbidity index, n(%)				
0	14,736 (24.2%)	13,158 (25.8%)	1578 (15.7%)	< 0.001
1–2	12,285 (20.2%)	9586 (18.8%)	2699 (26.9%)	
3–4	14,698 (24.1%)	11,994 (23.6%)	2704 (27.0%)	
> =5	19,224 (31.5%)	16,173 (31.8%)	3051 (30.4%)	
Length of stay, hours, median(IQR)	5.8 (2.0, 37.3)	4.8 (1.9, 36.0)	11.2 (3.6, 42.7)	< 0.001
Place of first admittance (Non-cardiac/CCU/Invasive centre)^a^	52,981/5267/2840	47,613/2357/1085	5368/2910/1755	
Final diagnosis				
Acute cardiac conditions, n(%):	2333 (3.8%)	1015 (2.0%)	1318 (13.1%)	< 0.001
Acute myocardial infarction:	1482 (2.4%)	428 (0.8%)	1054 (10.5%)	
STEMI	453 (0.7%)	113 (0.2%)	340 (3.4%)	
NSTEMI	1029 (1.7%)	315 (0.6%)	714 (7.1%)	
Other acute cardiovascular conditions:	851 (1.4%)	587 (1.1%)	264 (2.6%)	
UAP	181 (0.3%)	41 (0.1%)	140 (1.4%)	
Cardiac arrest	204 (0.3%)	193 (0.4%)	11 (0.1%)	
Ventricular tachycardia	53 (0.1%)	38 (0.1%)	15 (0.1%)	
Aortic dissection	114 (0.2%)	93 (0.2%)	21 (0.2%)	
Pulmonary Embolism	299 (0.5%)	222 (0.4%)	77 (0.8%)	
Other cardiovascular and non-cardiovascular conditions, n(%):	44,125 (72.2%)	40,478 (79.3%)	3647 (36.4%)	< 0.001
Certain infectious and parasitic diseases	1928 (3.2%)	1822 (3.6%)	106 (1.1%)	
Neoplasms	580 (0.9%)	559 (1.1%)	21 (0.2%)	
Endocrine, nutritional and metabolic diseases	1373 (2.2%)	1306 (2.6%)	67 (0.7%)	
Mental and behavioral disorders	1959 (3.2%)	1851 (3.6%)	108 (1.1%)	
Diseases of the nervous system	2004 (3.3%)	1962 (3.8%)	42 (0.4%)	
Diseases of the circulatory system (other than acute)	6332 (10.4%)	4598 (9.0%)	1734 (17.3%)	
Diseases of the respiratory system	6453 (10.6%)	5854 (11.5%)	599 (6.0%)	
Diseases of the digestive system	3405 (5.6%)	3097 (6.1%)	308 (3.1%)	
Diseases of the musculoskeletal system and connective tissue	1239 (2.0%)	1126 (2.2%)	113 (1.1%)	
Diseases of the genitourinary system	1853 (3.0%)	1760 (3.4%)	93 (0.9%)	
Injury, poisoning and certain other consequences of external causes	12,739 (20.9%)	12,587 (24.7%)	152 (1.5%)	
Other diagnoses^b^	4260 (7.0%)	3956 (7.7%)	304 (3.0%)	
No final diagnosis, n(%):	14,630 (23.9%)	9562 (18.7%)	5068 (50.5%)	< 0.001
Symptoms, signs and abnormal clinical and laboratory findings	8571 (14.0%)	6177 (12.1%)	2394 (23.9%)	
Medical observation and evaluation for suspected diseases and conditions or other reasons	6059 (9.9%)	3385 (6.6%)	2674 (26.7%)	

*GP* General practitioner, *IQR* interquartile range, *UAP* Unstable angina pectoris, *STEMI* ST-segment elevation myocardial infarction, *NSTEMI* non ST-segment elevation myocardial infarction

^a^ Place of first admittance (Non-cardiac department, including ED / CCU /Invasive Centre)

^b^Chapters containing < 1% of the total number of transports

### Main results

**Fig. 1:**
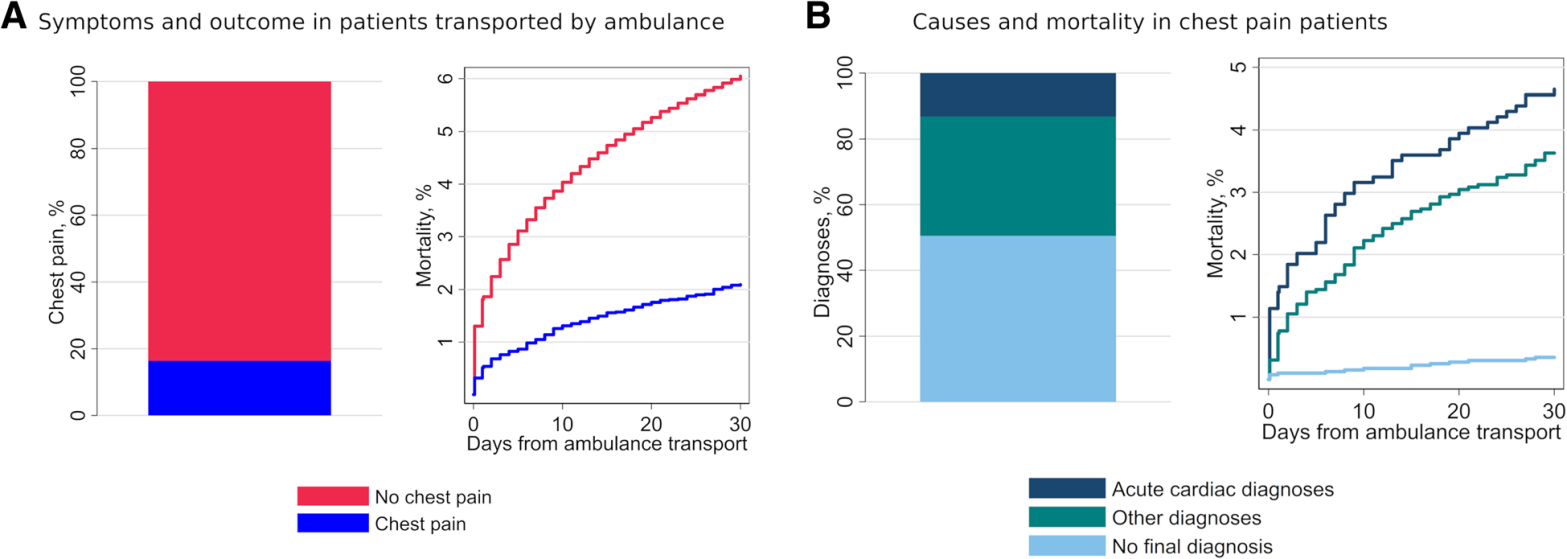
**a** Presence of chest pain and outcome in patients transported by ambulance. **b** Causes and outcome in chest pain patients divided in 3 groups: “Acute cardiac diagnoses”, “Other diagnoses” and “No diagnosis”. See Additional file 5 for raw values and categories (**a**) and time-to-event-or-censoring and censor status per patient (**b**)

Chest pain was the main symptom in 10,033 (16.4%) of ambulance transports. This equals eight transports due to chest pain per 1000 inhabitants per year in the Central Denmark Region. Thirty-day mortality rate was considerably lower in chest pain patients than in patients without chest pain, 2.1% (95%CI 1.8–2.4) compared to 6.0% (95%CI 5.7–6.2), Fig. 1a. After exclusion of patients requesting an ambulance due to conditions with very high risk of mortality (unconsciousness, cardiac arrest, suspected death, drowning, diving accidents, foreign matter blocking airway and GP-triaged patients dying within first admission day), the 30-day mortality in non-chest pain patients were still more than twice as high as in chest pain patients (4.7% (95%CI 4.5–5.0) vs 2.1% (95%CI 1.8–2.4)). Chest pain patients were more likely to receive a prehospital A-triage by the EMCC compared to non-chest pain patients (88% vs 38%), they had a higher frequency of prior AMI (16% vs 5%), and were more often triaged directly to a department of cardiology (46% vs 7%). Median LOS was more than twice as long in chest pain patients, compared to non-chest pain patients (11.2 vs 4.8 h) (*p* < 0.001 for all mentioned variables), see Table 1.

### Diagnostic patterns and outcome

A disease-specific diagnosis was established in 49.5% of chest pain patients (AMI 10.5%, other acute cardiovascular conditions 2.6%, and other diagnoses 36.4%) whereas 50.5% received no disease-specific diagnosis during index admission, Table 1. The rate of acute cardiac diagnoses assigned during the index admission, was higher in patients with chest pain compared to patients without chest pain (13% vs 2%) (*p* < 0.001), see Table 1. However, patients presenting with chest pain were also more often discharged without a disease-specific diagnosis (51% vs 19%) (*p* < 0.001). The 30-day mortality in chest pain patients was lowest in those not receiving a disease specific diagnosis during index admission (< 0.5%), Table 2 and Fig. 1b.

**Table 2:** Length of stay and 30-day mortality among GP- and 1–1-2 triaged chest pain patients according to ICD10-diagnosis

	Total transports, n	Median LOS, hours (Q1;Q3)	30-day Mortality, % (95%CI)
Number of transports	10,033	10.6 (3.6;41.3)	2.1% (1.8–2.4%)
Acute cardiac conditions	1318	88.0 (53.6;122.3)	4.6% (3.4–5.9%)
AMI	1054	91.0 (65.0;122.4)	4.0% (2.8–5.5%)
STEMI	340	93.0 (72.3;120.5)	3.5% (1.8–6.2%)
NSTEMI	714	88.4 (55.2;124.5)	4.2% (2.8–6.1%)
Other acute cardiovascular conditions:	264	65.2 (14.2;121.9)	7.0% (4.0–11.3%)
UAP	140	62.5 (28.6;108.1)	1.8% (0.2–6.4%)
Cardiac arrest	11	1.3 (1.0;123.3)	60.0% (26.2–87.8%)
Ventricular tachycardia	15	73.6 (48.6;209.1)	0.0% (0.0–30.8%)
Aortic dissection	21	137.4 (3.0;266.4)	21.1% (6.1–45.6%)
Pulmonary Embolism	77	69.1 (4.1;99.8)	4.6% (1.0–12.9%)
Other conditions:	3647	16.0 (3.9;53.1)	3.6% (2.9–4.4%)
Certain infectious and parasitic diseases	106	13.8 (2.4;132.3)	11.8% (5.6–21.3%)
Neoplasms	21	73.5 (3.0;265.2)	35.7% (12.8–64.9%)
Endocrine, nutritional and metabolic diseases	67	6.1 (3.0;32.7)	8.1% (1.7–21.9%)
Mental and behavioral disorders	108	2.7 (1.3;15.1)	0.0% (0.0–7.0%)
Diseases of the nervous system	42	5.0 (2.4;20.5)	3.4% (0.1–17.8%)
Diseases of the circulatory system (other than acute)	1734	20.6 (7.3;52.3)	2.5% (1.7–3.5%)
Diseases of the respiratory system	599	22.3 (3.1;93.0)	6.3% (4.2–9.0%)
Diseases of the digestive system	308	10.2 (2.6;53.7)	2.2% (0.7–5.1%)
Diseases of the musculoskeletal system and connective tissue	113	5.0 (2.0;8.9)	2.4% (0.3–8.2%)
Diseases of the genitourinary system	93	19.2 (5.0;70.3)	3.5% (0.4–12.1%)
Injury, poisoning and certain other consequences of external causes	152	2.9 (1.7;6.0)	3.2% (0.7–9.1%)
Other diagnoses^a^	304	10.0 (2.8;27.8)	2.4% (0.8–5.5%)
No final diagnosis:	5068	7.4 (3.0;14.8)	0.4% (0.2–0.6%)
Symptoms, signs and abnormal clinical and laboratory findings	2394	3.9 (2.0;8.6)	0.5% (0.2–0.9%)
Medical observation and evaluation for suspected diseases and conditions or other reasons	2674	10.3 (6.4;19.5)	0.2% (0.1–0.6%)

*GP* General practitioner, *UAP* Unstable angina pectoris, *AMI* Acute myocardial infarction, *STEMI* ST-segment elevation myocardial infarction, *NSTEMI* non ST-segment elevation myocardial infarction

^a^ICD-10 Chapters containing < 1% of the total number of transports

### Potential for downgrading EMS response

When including all chest pain patients into our multivariable model (Model 1), female gender, younger age, congestive heart disease, and chronic pulmonary disease were all associated with being discharged without an acute cardiac diagnosis. Age below 70 and no chronic pulmonary disease were all associated with surviving 30 days after a chest pain event, see Table 3.

**Table 3:** Predictors of mortality and discharge with an acute cardiac diagnosis in all chest pain patients

		30-day mortality			Acute cardiac diagnosis during index admission		
	Total^a^	Risk Ratio (95% CI)	*P*-value	Deaths, n(%)	Risk Ratio (95% CI)	*P*-value	Acute cardiac diagnoses, n(%)
Total	7635			160 (2%)			1140 (15%)
Gender							
Female	3485	1.0 (ref.)		74 (2%)	1.0 (ref.)		375 (11%)
Male	4150	1.06 (0.78;1.44)	0.721	86 (2%)	1.77 (1.58;1.99)	< 0.001	765 (18%)
Age-group							
< 30	344	1.0 (ref.)		2 (1%)	1.0 (ref.)		6 (2%)
30–49	1344	0.12 (0.01;1.37)	0.089	1 (%)	4.71 (2.09;10.62)	< 0.001	109 (8%)
50–59	1373	0.24 (0.03;1.68)	0.149	2 (%)	9.05 (4.05;20.21)	< 0.001	208 (15%)
60–69	1691	1.94 (0.46;8.23)	0.369	22 (1%)	11.77 (5.29;26.18)	< 0.001	326 (19%)
> =70	2883	6.33 (1.56;25.61)	0.010	133 (5%)	11.39 (5.13;25.32)	< 0.001	491 (17%)
Acute Myocardial Infarction (AMI)							
No prior AMI	6785	1.0 (ref.)		132 (2%)	1.0 (ref.)		1005 (15%)
Prior AMI	850	0.96 (0.63;1.46)	0.835	28 (3%)	0.96 (0.81;1.14)	0.677	135 (16%)
Diabetes (DM)							
No DM	6812	1.0 (ref.)		131 (2%)	1.0 (ref.)		1013 (15%)
DM	823	1.11 (0.74;1.66)	0.618	29 (4%)	0.97 (0.82;1.15)	0.758	127 (15%)
Congestive heart disease (CHD)							
No CHD	7003	1.0 (ref.)		128 (2%)	1.0 (ref.)		1065 (15%)
CHD	632	1.33 (0.88;2.02)	0.178	32 (5%)	0.69 (0.55;0.87)	0.002	75 (12%)
Chronic pulmonary disease (CPD)							
No CPD	6757	1.0 (ref.)		114 (2%)	1.0 (ref.)		1053 (16%)
CPD	878	1.9 (1.35;2.68)	< 0.001	46 (5%)	0.59 (0.48;0.72)	< 0.001	87 (10%)
Renal disease (RD)							
No or mild RD	7325	1.0 (ref.)		139 (2%)	1.0 (ref.)		1097 (15%)
Moderate to severe RD	310	1.7 (1.06;2.71)	0.027	21 (7%)	0.9 (0.68;1.2)	0.466	43 (14%)

^a^Individual patients, including only first transport in study period

When combining Model 1 with accompanying symptoms as predictors in the multivariable analysis (Model 2) in the subgroup of chest pain patients undergoing criteria-based dispatch, both absence of dyspnoea, absence of radiation/severe pain for > 5 min and absence of clammy skin/uncomfortable/nausea were associated with being discharged without a severe cardiac diagnosis. Absence of an accompanying symptom of non-responsiveness and dyspnoea were associated with surviving 30 days after a chest pain event, see Table 4.

**Table 4:** Predictors of mortality and discharge with a acute cardiac diagnosis, according to accompanying symptom in the subgroup of patients with chest pain undergoing criteria-based dispatch

		30-day mortality			Acute cardiac diagnosis during index admission		
	Total	Risk Ratio (95% CI)	*P*-value	Deaths, n(%)	Risk Ratio (95% CI)	*P*-value	Acute cardiac diagnoses, n(%)
Total	2634			69 (3%)			397 (15%)
Accompanying symptoms							
Non-responsive	9	9.79 (1.31;72.92)	0.026	1 (11%)	2.56 (0.74;8.86)	0.136	2 (22%)
Dyspnoea	566	2.15 (1.03;4.51)	0.043	26 (5%)	1.8 (1.27;2.56)	0.001	82 (14%)
Radiation/severe pain> 5 min	1068	1.25 (0.59;2.62)	0.558	26 (2%)	2.06 (1.5;2.84)	< 0.001	183 (17%)
Clammy skin/Uncomfortable/Nausea	488	0.8 (0.3;2.12)	0.653	7 (1%)	2.12 (1.51;3.0)	< 0.001	90 (18%)
Other or no	503	1.0 (ref.)		9 (2%)	1.0 (ref.)		40 (8%)

Female gender, younger age, chronic pulmonary disease, absence of dyspnoea, absence of radiation/severe pain for > 5 min and absence of clammy skin/uncomfortable/nausea were associated with the combined endpoint of being discharged without an acute cardiac diagnosis and surviving 30 days after chest pain event.

When applied in a diagnostic model, Model 2 identified 14% of the patients eventually discharged without an acute cardiac diagnosis and surviving 30 days after a chest pain event, with a sensitivity of 99%, a negative predictive value (NPV) of 98%, and a negative likelihood-ratio of 0.08. Thirty-day mortality in these patients was 0.7%.

## Limitations

This large study is based on all registrations of ambulance transports in the Central Denmark Region in a 1 year period, combined with diagnoses and vital status from validated national registers. However, we did exclude 6% of the cohort due to missing data on either symptom or diagnosis, and selection bias cannot be excluded. Available characteristics, diagnoses and outcome in these patients are presented in Additional file 2. In short, we see a very low incidence of AMI and other acute cardiac diagnoses (< 2%) in the symptom-missing patients and likewise a low incidence of chest pain (6%) in the diagnose-missing patients. Thirty-day mortality in the symptom-missing group is not different from the mortality rate in the overall cohort (6.2% vs 5.4%). Mortality in the diagnose-missing group was slightly higher (6.7%) but as mentioned, this group includes persons dying during ambulance transport or found dead, and subsequently transported to hospital for confirmation of death by a doctor, which can explain the observed higher mortality. We conclude that the risk of selection bias due to exclusion of these patients is very low. There is a risk of immortal time bias in our regression models, since we only include patients surviving to hospital admission. However, in prehospital terminated transports, 30-day mortality among chest pain patients was as low as 3.2%, disputing the possible immortal time bias.

In mortality analysis 70 patients are lost-to-follow-up, meaning that they are registered as inactive in the Danish civil registration system. However, only 13 of these where registered as inactive within 30 days after their chest pain event, while the remaining 57 where inactive at the time of their chest pain event (e.g. migrated or without residence in Denmark).

The study is limited by its retrospective design, which affects both the identification of symptoms (if specific information is missing) and potentially biases the effect of patient characteristics, symptoms and comorbidity, since we cannot definitely differentiate between correlation and causality. The identification of chest pain could be limited by the fact that only 37% of patients were triaged using symptom-based dispatch. Instead, symptom-identification relied on automatized text search in notes from electronical prehospital patient records. This method may be sensitive to spelling and abbreviation of words in the records. We sought to compensate for this by including several spellings and known abbreviations of each word, by excluding terms describing rejection of chest pain symptoms and by manual validation of the method in > 5% of the cohort. The automatized text search showed very good agreement with the manual reviews (kappa = 0.95), Additional file 3.

## Discussion

This retrospective study in a large cohort of patients being transported by ambulance has three major findings. Firstly, chest pain is a very common reason for acute ambulance transport and hospital admission, accounting for one in six emergent ambulance transports. Secondly, acute ambulance transport in chest pain patients is associated with a high degree of overtriage with more than 50% of transported chest pain patients discharged without any diagnosis of disease and with a very low (< 0.5%) 30 day-mortality risk. Thirdly, it may be possible to identify a very-low-risk group already at the time of emergency call based on patient characteristics and symptomatology, indicating a potential for downgrading the response in selected chest pain patients.

The rate of chest pain patients transported by ambulance was comparable to the chest pain rate found in recent Swedish and Danish studies, but substantially higher than earlier reported in studies from Norway and the UK [2, 20–23]. The most likely reason for this higher rate is a temporal increase in chest pain cases also reported in other studies [23, 24]. This increase may be explained by an increased focus in the general population and in health care professionals on the need for acute assessment in cases with chest pain. Both chest pain and AMI rates in the present study are also comparable to rates previously reported from European and American ED cohorts [1, 25].

When compared to patients without chest pain, chest pain patients were more often diagnosed with an acute cardiac condition including AMI (13% vs 2%), but also more often discharged with no final diagnosis (51% vs. 19%). This is a paradox and reflects on one hand the relevance of chest pain as a symptom of acute cardiovascular disease, but on the other hand also an overtriage of chest pain patients compared to patients presenting with other symptoms. The burden added to the EMS by chest pain patients is exaggerated by the fact that 88% of these patients were allocated a “grade A” response, thus representing 31% of all “grade A” responses. The decision to dispatch a “grade A” response is currently not based on an individual risk-estimation, but rather a “no miss” strategy, where the majority of patients with symptoms with even the slightest risk of AMI, are offered the highest level of care. The result is a significant overtriage, where more than half of all chest pain patients are discharged with no disease specific diagnosis and extremely low 30-day mortality below 0.5%. This advocates the use of a systematic differentiation between high-risk chest pain patients with urgent need of treatment and very low-risk chest pain patients without need for hospital admission. In the EMCC, this may be achieved by including patient characteristics and symptoms (readily accessible at the time of emergency call) in a risk assessment tool.

In explorative analyses on the present cohort (Tables 3 and 4) female gender, younger age, chronic pulmonary disease, absence of dyspnoea, absence of radiation/severe pain for > 5 min and absence of clammy skin/uncomfortable/nausea were associated with being discharged without an acute cardiac diagnosis and surviving 30 days after a chest pain event. Applied in a diagnostic model for the composite endpoint of no acute cardiac diagnosis during index admission and surviving 30 days from chest pain event, Model 2 presented acceptable sensitivity, NPV and negative likelihood ratio. However, since these data were retrospective, they were not gathered with this in mind. Thus, though risk assessment using patient characteristics and clinical presentation/ accompanying symptoms seems promising, safety of such a strategy should be further refined and explored in prospective studies. Future studies should aim at confirming that an estimated very low risk justifies a lower prehospital dispatch priority and potentially prehospital rule out of AMI and other acute conditions already at medical emergency call.

Clinical implementation of a risk assessment strategy may further unfold as the patient pass through the EMS system. Early rule-out of AMI in the ED using patient characteristics and clinical presentation in combination with high-sensitive troponin has been validated in several recent studies [26–28]. Even earlier rule-out of AMI could be achieved by combining high-sensitive troponin with copeptin, maybe even in the prehospital setting, when equipment for point-of-care copeptin measurement is developed [29–31]. The prerequisites for this dual marker approach are examined in an ongoing randomized controlled study [32]. With a risk assessment tool used at time of emergency call, resource downgrading after ambulance dispatch may be based on incorporation of subsequent clinical assessment, ECG-findings and biomarker results in a stepwise, individual risk assessment model, even before reaching hospital.

## Conclusion

Chest pain is the reason for 16% of all ambulance transports. AMI is only diagnosed in 11% of chest pain patients. The mortality in chest pain patients is lower than in other patients transported to hospital by ambulance, and particularly low in chest pain patients not receiving a diagnosis of disease during index admission. Structured risk assessment using patient characteristics and symptoms seems to hold a potential for early resource downgrading in low risk chest pain patients, already in the prehospital setting.

## Additional files

Additional file 1: Patient flowchart. Flowchart of study patients. (PDF 121 kb)

Additional file 2: Prehospitally terminated transports and transports missing symptoms or final diagnosis. Characteristics, symptoms and diagnoses in transports terminated prehospitally, or in which either symptoms or final diagnosis is missing. (PDF 424 kb)

Additional file 3: Identification of chest pain. Details on Danish Index codes indicating chest pain and the automatic text search and validation of this. (PDF 605 kb)

Additional file 4: ICD-10 codes. ICD-10 diagnoses used to categorize final diagnosis, calculate Charlson Comorbidity Index (CCI), and to identify known comorbidity. (PDF 531 kb)

Additional file 5: Data for Fig. 1. Raw values and categories (Fig. 1a) and time-to-event-or-censoring and censor status per patient (Fig. 1b) (XLS 7940 kb)

## Data Availability

The datasets generated and/or analysed during the current study are not publicly available owing to protection of personal data, but data and statistical codes are available from the corresponding author on reasonable request and with permission of relevant Danish authorities.

## Abbreviations

AMI: Acute myocardial infarction
CCI: Charlson Comorbidity Index
CCU: Coronary Care Unit
CI: Confidence interval
ECG: Electrocardiogram
ED: Emergency Department
EMCC: Emergency Medical Communication Centres
EMS: Emergency Medical Service
GP: General Practitioner
IQR: Interquartile ranges
LOS: Length of stay
NPV: Negative predictive value
