# Supplementary figures and images for: Chest pain in the ambulance; prevalence, causes and outcome - a retrospective cohort study
Source: Scand J Trauma Resusc Emerg Med. 2019 Aug 29;27:84. doi: 10.1186/s13049-019-0659-6 (PMC6716930; doi:10.1186/s13049-019-0659-6)

# Patient flowchart

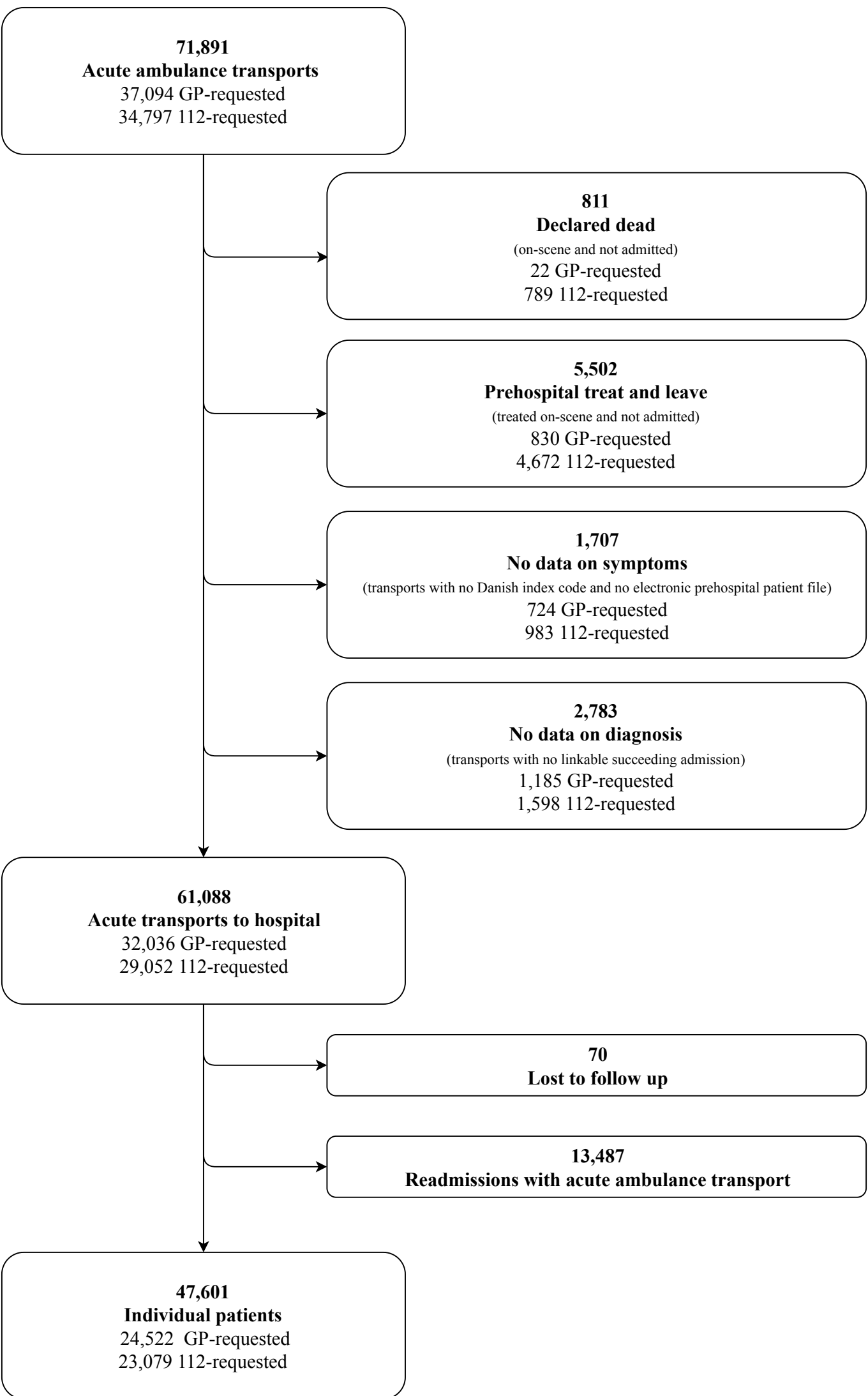

Supplement: Supplementary file 1 — Patient flowchart. Flowchart of study patients. (PDF 121 kb) [file 13049_2019_659_MOESM1_ESM.pdf]
