# Supplementary material for: Chest pain in the ambulance; prevalence, causes and outcome - a retrospective cohort study
Source: Scand J Trauma Resusc Emerg Med. 2019 Aug 29;27:84. doi: 10.1186/s13049-019-0659-6 (PMC6716930; doi:10.1186/s13049-019-0659-6)
Supplement: Supplementary file 2 — Prehospitally terminated transports and transports missing symptoms or final diagnosis. Characteristics, symptoms and diagnoses in transports terminated prehospitally, or in which either symptoms or final diagnosis is missing. (PDF 424 kb) [file 13049_2019_659_MOESM2_ESM.pdf]

Additional file 2: Prehospitally terminated transports and transports missing symptoms or final diagnosis

Prehospitally terminated transports include transports of patients treated on-scene and left at home and patients declared dead and not transported to the morgue.

In the transports missing information on symptoms, we expect the information to be missing at random, as no association between chest pain and prehospital registration of symptoms are likely.

Transports with missing linkage between transport and admission (and thereby final diagnosis), includes transports of persons dying during ambulance transport and persons found dead and transported to hospital for legal or logistic reasons. Excluding these deceased patients, we expect the diagnoses to be missing at random or if non-random, to be less likely in patients receiving a diagnosis of disease, rather than in patients with "No final diagnosis" (ICD-10 diagnosis of "Rxx.x", "Z03.x" or "Z04.x").

**Characteristics, symptoms and diagnoses in transports terminated prehospitally, missing either symptoms or final diagnosis**				
		Prehospital terminated transports	LPR missing	Symptom missing
Acute ambulance transports, n(%)		6,313	2,783	1,707
Way of ambulance request, n(%)	GP	852 (13.5%)	1185 (42.6%)	724 (42.4%)
	112	5461 (86.5%)	1598 (57.4%)	983 (57.6%)
Pre-hospital triage, n(%)	A	4205 (66.7%)	1531 (55.5%)	877 (54.5%)
	B	2047 (32.5%)	971 (35.2%)	337 (20.9%)
	C	50 (0.8%)	259 (9.4%)	396 (24.6%)
Male gender, n(%)		3534 (56.0%)	1449 (52.1%)	859 (50.3%)
Age, median (IQR)		55 (34, 72)	56 (35, 74)	62 (38, 76)
Diabetes, n(%)		1025 (19.2%)	270 (10.3%)	184 (10.8%)
Prior AMI, n(%)		277 (5.2%)	122 (4.7%)	92 (5.4%)
Charlson comorbidity index, n(%)	0	1414 (26.5%)	804 (30.7%)	513 (30.1%)
	1-2	1267 (23.7%)	632 (24.1%)	334 (19.6%)
	3-4	1152 (21.6%)	565 (21.5%)	344 (20.2%)
	>=5	1507 (28.2%)	621 (23.7%)	516 (30.2%)
30-day mortality		988 (15.7%)	188 (6.8%)	106 (6.2%)
1-day mortality		878 (13.9%)	111 (4.0%)	19 (1.1%)
Chest pain		620 (%)	232 (8.7%)	
Serious cardiac conditions				33 (1.9%)
AMI				13 (0.8%)
STEMI				8 (0.5%)
NSTEMI				5 (0.3%)
Other serious cardiovascular conditions:				18 (1.1%)
UAP				2 (0.1%)
Cardiac arrest				9 (0.5%)
Aortic dissection				2 (0.1%)
Pulmonary Embolism				7 (0.4%)
Other conditions:				1,308 (76.6%)
Certain infectious and parasitic diseases				41 (2.4%)
Neoplasms				15 (0.9%)
Endocrine, nutritional and metabolic diseases				39 (2.3%)
Mental and behavioral disorders				68 (4.0%)
Diseases of the nervous system				54 (3.2%)
Diseases of the circulatory system				124 (7.3%)
Diseases of the respiratory system				190 (11.1%)
Diseases of the digestive system				90 (5.3%)
Diseases of the musculoskeletal system and connective tissue				43 (2.5%)
Diseases of the genitourinary system				41 (2.4%)
Injury, poisoning and certain other consequences of external causes				487 (28.5%)
Other diagnoses				116 (6.8%)
No final diagnosis:				366 (21.4%)
Symptoms, signs and abnormal clinical and laboratory findings				193 (11.3%)
Medical observation and evaluation for suspected diseases and conditions or other reasons				173 (10.1%)
