# Supplementary material for: Chest pain in the ambulance; prevalence, causes and outcome - a retrospective cohort study
Source: Scand J Trauma Resusc Emerg Med. 2019 Aug 29;27:84. doi: 10.1186/s13049-019-0659-6 (PMC6716930; doi:10.1186/s13049-019-0659-6)
Supplement: Supplementary file 3 — Identification of chest pain. Details on Danish Index codes indicating chest pain and the automatic text search and validation of this. (PDF 605 kb) [file 13049_2019_659_MOESM3_ESM.pdf]

Additional file 3: Identification of chest pain

Chest pain/ Chest discomfort were identified from prehospital patient records and from "Danish Index for Emergency Care" codes:

From full-text searches the prehospital patient record notes written by the paramedics:
if the notes included any variety of the terms: "chest pain" or "chest discomfort" and without any terms rejecting chest pain or chest discomfort.

Danish terms searched:
" knugen (midt/centralt) (hen)over/(for/i) (venstre/højre side af) bryst(et)/thorax/brystkasse(n)",
" smerte(r) (midt/centralt) (hen)over/for/i) (venstre/højre side af) bryst(et)/thorax/brystkasse(n)",
"ubehag (midt/centralt) (hen)over/for/i) (venstre/højre side af) bryst(et)/thorax/brystkasse(n)",
"ondt (midt/centralt) (hen)over/for/i) (venstre/højre side af) bryst(et)/thorax/brystkasse(n)",
" tryk(ken) (midt/centralt) (hen)over/for/i) (venstre/højre side af) bryst(et)/thorax/brystkasse(n)",
"retrosternal(e) smerte(r)",
"bryst( )smerte(r)",
"angina",

The text search included several spellings and abbreviations of each word.

We excluded any terms describing rejection of chest pain/chest discomfort symptoms.

Validation:

To refine and validate the text search algorithm, we performed manual validation in > 5% of the cohort:

1) manual validation of 500 prehospital patient records by 1 author to develop the text search algorithm
2) manual validation of 2000 prehospital patient records by 6 doctors experienced in evaluating chest pain patients, to refine the text search algorithm
3) manual validation of 1000 prehospital patient records by 3 doctors experienced in evaluating chest pain patients, for final validation.

Result of validation:

	A	B	Total
A	490	10	**500**
B	15	485	**500**
**Total**	**505**	**495**	**1000**

Number of observed agreements: 975 (97.5%)
Number of agreements expected by chance: 500 (50.5%)
Kappa= 0.95 (=Very good agreement)

Most common reason for misclassification was mis-spellings or abnormal discriptions of chest pain/discomfort.

2) <u>Danish Index for Emergency Care (DIEC) codes, reflecting chest pain and accompanying symptoms:</u>
- assigned by the emergency medical dispatcher at the time of call

DIEC-code	English term	Danish term	Accompanying symptom
A.10.01	Non-responsive to speech or pain stimuli.	Reagerer ikke på tilråb eller smertestimuli.	1- Non-responsive
A.10.02	Chest pain and fainting.	Ondt i brystet og er ved at besvime.	5- Other
A.10.03	Severe pain in center of the chest for more than 5 min.	Stærke smerter midt i brystet i mere end 5 min.	3-Radiation/severe pain>5min
A.10.04	- difficulty breathing	- vejrtrækningsbesvær	2- Dyspnea
A.10.05	- Uncomfortable, nausea	- utilpas, kvalme	4 - clammy skin /Uncomfortable/nausea
A.10.06	- pale, clammy skin	- bleg, klam hud	4 - clammy skin /Uncomfortable/nausea
A.10.07	- radiation of pain to the jaw / shoulder / arm / back	- udstråling af smerterne til kæbe/skulder/arm/ryg	3-Radiation/severe pain>5min
A.10.08	- suddenly feeble in arms	- pludselig kraftesløs i armene	5- Other
A.10.09	- ECG showing myocardial infarction (STEMI)	- EKG som viser hjerteinfarkt (STEMI)	5- Other
A.10.10	- transient effect of nitroglycerin.	- kun forbigående virkning af nitroglycerin.	5- Other
A.10.11	Possible serious heart condition, presenting with atypical symptoms, including palpitations, accompanied by severe constitutional symptoms.	Mulig alvorlig hjerteproblem med atypiske symptomer, herunder hjertebanken ledsaget af alvorlige almensymptomer.	5- Other
A.10.14	Chest pain - otherwise unaffected.	Smerter i brystet - ellers upåvirket.	5- Other
C.10.01	The pain is not particularly severe, patient is feeling OK.	Smerterne er ikke specielt stærke og pt. føler sig OK.	5- Other
C.10.02	Persistent effect of 1-4 nitroglycerin tablets / sprays.	Vedvarende god virkning af 1-4 nitroglycerintabletter/spraydoser.	5- Other
C.10.03	The pain/discomfort is located superficially, in the lateral part of the chest.	Smerterne/ubehaget sidder overfladisk i siden af brystkassen.	5- Other
E.10.01	Pain only in association with deep inhalation or movement	Smerter kun ved dyd indånding eller ved bevægelse	5- Other
A.28.03	Chest pain and breathing difficulties.	Brystsmerter og vejrtrækningsproblemer.	2- Dyspnea
