# Supplementary material for: Chest pain in the ambulance; prevalence, causes and outcome - a retrospective cohort study
Source: Scand J Trauma Resusc Emerg Med. 2019 Aug 29;27:84. doi: 10.1186/s13049-019-0659-6 (PMC6716930; doi:10.1186/s13049-019-0659-6)
Supplement: Supplementary file 4 — ICD-10 codes. ICD-10 diagnoses used to categorize final diagnosis, calculate Charlson Comorbidity Index (CCI), and to identify known comorbidity. (PDF 531 kb) [file 13049_2019_659_MOESM4_ESM.pdf]

Additional file 4: ICD-10 codes

Categorization of final diagnosis (ICD-10,version 2016):

Serious cardiac conditions

AMI: I21.x
STEMI: I21.0, I21.1, I21.3
NSTEMI: AMI, but not STEMI
UAP: I20.0
Cardiac arrest: I46.x
Atrioventricular block: I44.1, I44.2
Ventricular Tachycardia: I47.0, I47.2, I47.2, I47.2
Aortic dissection: I71.0, I71.1, I71.3, I71,5 , I71.8
Pulmonary Embolism: I26.x
Pneumothorax: J93.x

Other diagnoses according to ICD-10 chapters (expected to above mentioned)

I Certain infectious and parasitic diseases
A00-A09, A15-A28, A30-A89, A92-A99, B00-B09, B15-B83, B85-B99
II Neoplasms
C00-C97, D00-D48
III Diseases of the blood and blood-forming organs and certain disorders involving the immune mechanism
D50-D53, D55-D77, D80-D89
IV Endocrine, nutritional and metabolic diseases
E00-E07, E10-E16, E20-E35, E40-E46, E50-E68, E70-E90
V Mental and behavioral disorders
F00-F48, F50-F99
VI Diseases of the nervous system
G00-G14, G20-G26, G30-G32, G35-G37, G40-G47, G50-G64, G70-G73, G80-G83, G90-G99
VII Diseases of the eye and adnexa
H00-H06, H10-H13, H15-H22, H25-H28, H30-H36, H40-H59
VIII Diseases of the ear and mastoid process
H60-, H65-H75, H80-H83, H90-H95
IX Diseases of the circulatory system
I00-I02, I05-I15, I20-I25, I26-I28, I30-I52, I60-I89, I95-I99
X Diseases of the respiratory system
J00-J06, J09-J18, J20-J22, J30-J47, J60-J70, J80-J86, J90-J99
XI Diseases of the digestive system
K00-K14, K20-K31, K35-K38, K40-K46, K50-K52, K55-K67, K70-K77, K80-K87, K90-K93
XII Diseases of the skin and subcutaneous tissue
L00-L08, L10-L14, L20-L30, L40-L45, L50-L75, L80-L99
XIII Diseases of the musculoskeletal system and connective tissue
M00-M25, M30-M36, M40-M54, M60-M99
XIV Diseases of the genitourinary system
N00-N08, N10-N23, N25-N51, N60-N64, N70-N77, N80-N99
XV Pregnancy, childbirth and the puerperium
O00-O08, O10-O16, O20-O48, O60-O75, O80-O92, O94-O99
XVI Certain conditions originating in the perinatal period
P00-P08, P10-P15, P20-P29, P35-P39, P50-P61, P70-P78, P80-P83, P90-P96
XVII Congenital malformations, deformations and chromosomal abnormalities
Q00-Q07, Q10-Q18, Q20-Q28, Q30-Q45, Q50-Q56, Q60-Q99
XIX Injury, poisoning and certain other consequences of external causes
S00-S99, T0-T88, T90-T98
XX External causes of morbidity and mortality
V01-X59, X60-X84, X85-Y36, Y40-Y98
XVIII Symptoms, signs and abnormal clinical and laboratory findings, not elsewhere classified
DR252A, DR258A, DR252B, DR40,DR47, DR55, DR57, DR99, DR100A
XXI Factors influencing health status and contact with health services
Z00-Z13, Z20-Z65, Z70-Z76, Z80-Z99 (other than Z03 & Z04)

No final diagnosis

XVIII Symptoms, signs and abnormal clinical and laboratory findings, not elsewhere classified
R00-R23, R25-R29-R99 (other than DR252A, DR258A, DR252B, DR40,DR47, DR55, DR57, DR99, DR100AXXI)
XXI Factors influencing health status and contact with health services
Z03 -Z04

ICD-10 diagnoses used for calculation of Charlson comorbidity index:

Acute Myocardial Infarction
I21, I22, I23
Congestive Heart Failure
I50, I11.0, I13.0, I13.2
Peripheral Vascular Disease
I70, I71, I72, I73, I74, I77
Cerebrovascular Disease
G45, G46, I60, I61, I62, I63, I64, I65, I66, I67, I68, I69
Dementia
F00, F01, F02, F03, G30, F051
Chronic Pulmonary Disease
J40, J41, J42, J43, J44, J45, J46, J47, J60, J61, J62, J63, J64, J65, J66, J67, J68.4, J70.1, J70.3, J84.1, J92.0, J96.1, J98.2, J98.3
Rheumatologic Disease (Connective Tissue Disease)
M05, M06, M08, M09, M30, M31, M32, M33, M34, M35, M36, 86
Peptic Ulcer Disease (Ulcer disease)
K25, K26, K27, K28, K22.1
Mild Liver Disease
B18, K71, K73, K74, K70.0, K70.1, K70.2, K70.3, K70.9, K76.0
Diabetes I and II
E10.0, E10.1, E10.9, E11.0, E11.1, E11.9
Hemiplegia
G81, G82
Moderate to severe renal disease
I12, I13, N00, N01, N02, N03, N04, N05, N07, N11, N14, N17, N18, N19, Q61
Diabetes with end organ
E10.2, E10.3, E10.4, E10.5, E10.7, E10.8, E11.2, E11.3, E11.4, E11.5, E11.6, E11.7, E11.8
Any tumor
C00, C01, C02, C03, C04, C05, C06, C08, C09, C10, C11, C12, C13, C14, C15, C16, C17, C18, C19, C07, C20, C21, C22, C23, C24, C25, C26, C30, C31, C32, C33, C34, C37, C38, C39, C40, C41, C43, C44, C45, C46, C47, C48, C49, C50, C51, C52, C53, C54, C56, C57, C58, C60, C61, C62, C63, C64, C65, C66, C67, C68, C69, C55, C70, C71, C72, C73, C74, C75
Leukemia
C91, C92, C93, C94, C95
Lymphoma
C81, C82, C83, C84, C85, C88, C90, C96
Moderate to severe liver disease
B15.0, B16.0, B16.2, B19.0, K70.4, K76.6, K72, I85
Metastatic solid tumor
C76, C77, C78, C79, C80
AIDS
B21, B22, B23, B24
